# Advanced Biofuels Based on Fischer–Tropsch Synthesis for Applications in Diesel Engines

**DOI:** 10.3390/ma14113077

**Published:** 2021-06-04

**Authors:** Jan Jenčík, Vladimír Hönig, Michal Obergruber, Jiří Hájek, Aleš Vráblík, Radek Černý, Dominik Schlehöfer, Tomáš Herink

**Affiliations:** 1 Department of Chemistry, Faculty of Agrobiology, Food and Natural Resources, Czech University of Life Sciences Prague, Kamýcká 129, 165 00 Prague, Czech Republic; jencikj@af.czu.cz (J.J.); obergruber@af.czu.cz (M.O.); jirihajek@af.czu.cz (J.H.); 2 ORLEN UniCRE a.s., 436 01 Litvínov, Czech Republic; ales.vrablik@orlenunicre.cz (A.V.); radek.cerny@orlenunicre.cz (R.Č.); dominik.schlehofer@orlenunicre.cz (D.S.); tomas.herink@unipetrol.cz (T.H.)

**Keywords:** Fischer–Tropsch synthesis, biofuel, biodiesel, alternative fuels, standardization, waste materials

## Abstract

This paper focuses on the evaluation of the fuel properties of Fischer–Tropsch diesel blends with conventional diesel. Incorporating this advanced fuel into conventional diesel production will enable the use of waste materials and non-food materials as resources, while contributing to a reduction in dependence on crude oil. To evaluate the suitability of using Fischer–Tropsch diesel, cetane number, cetane index, CFPP, density, flash point, heat of combustion, lubricity, viscosity, distillation curve, and fuel composition ratios using multidimensional GC × GC-TOFMS for different blends were measured. It was found that the fuel properties of the blended fuel are comparable to conventional diesel and even outperform conventional fuel in some parameters. All measurements were performed according to current standards, thus ensuring the repeatability of measurements for other research groups or the private sector.

## 1. Introduction

Renewable energy sources can be converted directly into liquid fuels, generally named “biofuels”. Renewable energy sources cover a broad raw material base, including cellulosic biomass (fibrous and inedible parts of plants), waste materials, algae, and biogas [1]. As defined in Directive 2001/77/EC, biomass means “the biodegradable fraction of products, waste, and residues from agriculture (including vegetal and animal substances), forestry and related industries, as well as the biodegradable fraction of industrial and municipal waste”. New types of biofuels complementing the fuel market should meet the following criteria [2]:

- High quality and higher environmental aspects than the existing first generation biofuels;
- Usage in existing engines without any modifications;
- Usage should not affect the engine performance;
- Guaranteed miscibility with petroleum fuels in a defined proportion;
- Easy integration into the current fuel market;
- Production from waste sources is preferred.

Renewable Energy Directive II 2018/2001/ES (RED II) on the promotion of the use of energy from renewable sources was created for clarity due to Directive 2009/28/ES (RED I) being recast several times. Each Member State shall ensure, from 1 January 2021, at least 70% savings in greenhouse gas emissions from the use of liquid and gaseous fuels from renewable sources of non-biological origin used in the transport sector. Furthermore, from 1 January 2021, the share of energy from renewable sources in the gross final consumption of energy should be less than the basic share of energy from renewable sources in that year, as set out in the third column of the table in Annex I, Part A. For example, for the Czech Republic, it is set at 13%. Furthermore, the Directive sets out that within the share of final energy consumption in transport in each Member State, the share of advanced biofuels and biogas must be at least 0.2% in 2022, at least 1% in 2025, and at least 3.5% in 2030. Such mandatory national targets are consistent with the objective of at least a 32% share of energy from renewable sources in the gross final consumption of energy in 2030 in the European Union. Moreover, there are defined rules for calculating the share of energy from renewable sources in transport, with multiple associated rules listed as counting the energy content of biofuels made from raw materials. There is also a standardized formula for calculating greenhouse gas savings for fuels produced from biomass, including their given percentages [2].

The RED II Directive also strictly defines the sustainability criteria for all biofuels, bioliquids, and biomass fuels. These criteria deal with the origin of input material, production method, and greenhouse gas emission savings. Only those fuels that comply with these rules will be able to be distributed and sold commercially in the future.

### 1.1. First-Generation Biofuels

These biofuels are produced from biomass suitable for food production, and according to the Life cycle assessment (LCA) analysis, they show a low balance of CO_2_ production during the entire life cycle. First-generation biofuels have the potential to reduce CO_2_ emissions by about 50% compared to fossil fuels [3]. The raw materials for the production of first-generation biofuels include grain, corn, starch, sugar beet, and sugar cane. From these raw materials, bioethanol can be produced by fermentation and refining. Alternatively, biobutanol can be produced by the catalytic conversion of bioethanol or BioETBE (bioethyl tert-butyl ether), which is produced from the addition of bioethanol to isobutane. Fatty acid methyl esters (FAME), made from oil pressed from oilseeds such as palm oil, sunflower oil, and rapeseed oil, are used to produce an alternative to diesel [2,4].

### 1.2. Second-Generation Biofuels

These biofuels are produced from non-food and waste biomass such as forest biomass, including mining residues, agricultural waste (straw, hay, corn, rape, and other residues), energy plants (pterosaurs, sorghum, sorrel, etc.), and biological waste from households, such as used frying oil, waste animal fat, and municipal waste [5,6,7]. Second-generation energy crops have a significantly higher transformation potential for biofuels than the first generation [8]. However, the technological process is much more complex and demanding than the fermentative production of ethanol or the esterification of oils [9]. The conversion ratio is usually 5 tons of biomass can produce 1 ton of biofuel. Second-generation biofuels have the potential to reduce CO_2_ emissions by up to 90% compared to fossil fuels. According to the LCA analysis, second-generation biofuels show a significant positive difference in the balance of CO_2_ production during their life cycle [10,11].

Biofuels produced by these raw materials include bioethanol from lignocellulosic biomass, biomethanol, or alternative gasoline as a product of the catalytic conversion of synthesis gas, biobutanol from bioethanol, and diesel fuel produced by hydrogenation or transesterification of non-food or used cooking oils. However, from the point of view of circular economics, the production of synthetic fuels formed after pyrolysis of waste plastics into pyrolysis oil [12,13,14,15,16], or the conversion of synthesis gas to Fischer–Tropsch synthesis products, seems most promising [17].

### 1.3. Third-Generation Biofuels

Third-generation biofuels are liquid and gaseous biofuels obtained mainly from oil-producing algae in bioreactors. The biomass production yield is, in general, specified as 5 to 10 times higher than for land-based plants [18]. In short, algae only need sun, heat, CO_2_, and nutrients to produce oil droplets. The oil is processed into the appropriate fuel by known methods [18]. The lipid composition and starch content vary between microalgae and cyanobacterial species. Not all types of algae are useful for the production of biofuels. Some species are more suitable for the production of biofuels because they can naturally biosynthesize and accumulate significant amounts of intracellular lipids and starch. These properties can be further improved by genetic modification [19]. For example, the genetically modified *Chlorella vulgaris* mutant (UV715) contains up to 67% oil [20].

### 1.4. Fischer–Tropsch Synthesis

The Fischer–Tropsch synthesis (FT) is a catalyzed polymerization process operating on the surface of a heterogeneous catalyst that uses hydrocarbon monomers formed by the hydrogenation of adsorbed CO to produce both short- and long-chain hydrocarbons, and it has a wide range of applications [21]. It is thus a process in which carbon monoxide and hydrogen are converted by a catalytic reaction to a mixture of substances consisting mainly of n-alkanes (linear hydrocarbons) according to Equation (1), olefins according to Equation (2), and, to lesser extent, alcohols according to Equation (3). The description of the reactions taking place in the Fischer–Tropsch synthesis is as follows [22]:

(1) $$\left(2n+1\right)H_{2}+\mathrm{nCO}\to C_{n}H_{2n+2}+{\mathrm{nH}}_{2}O$$

(2) $$2{\mathrm{nH}}_{2}+\mathrm{nCO}\to C_{n}H_{2n}+{\mathrm{nH}}_{2}O$$

(3) $$2{\mathrm{nH}}_{2}+\mathrm{nCO}\to C_{n}H_{2n+1}\mathrm{OH}+{\mathrm{nH}}_{2}O$$

The physicochemical properties of synthetic and conventional liquid fuels are almost comparable. With some properties, synthetic fuels are significantly better (cetane number, very low content of aromatics, do not contain sulfur, nitrogen, heavy metals, asphaltenes, or salts). At present, as raw materials for the production of synthesis gas (CO + H_2_) [23], which is a raw material for the production of synthetic motor liquid fuels. Generally, regarding the technology of producing synthetic fuels, this method is referred to as an XTL. Depending on the source of the input raw material, “X” is replaced by the first letter of the given source [22]:

- GTL—natural gas;
- BTL—gasified biomass;
- CTL—coal dust.

**Figure 1:**
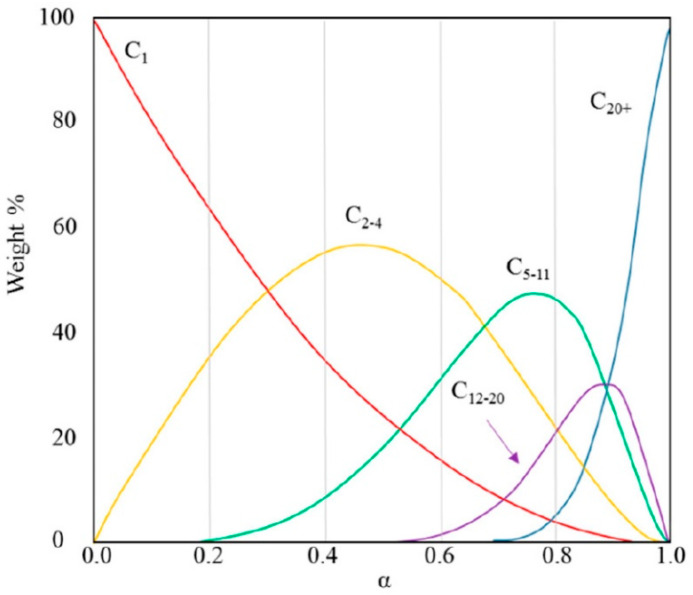
Dependence of quantity and type of products on the probability of chain growth [26].

Compared to conventional petroleum processing, products of Fischer–Tropsch synthesis do not contain salts, heavy metals, sulfur, nitrogen, or aromatic compounds. As a result, this technology has the potential to become a suitable technology for biomass processing. However, one disadvantage is the higher operating cost compared to the cost of processing oil [21]. There are various models for controlling Fischer–Tropsch synthesis with a focus on the production of hydrocarbons of the desired products. The most widespread and simplest model is the mathematically described distribution model of polymerization called Anderson–Schulz–Flory, also called “ASF-plot”. In this distribution model, FT synthesis is modeled as an addition polymerization reaction with a probability of α chain growth [24]. The resulting product distribution is shown as a graph of the logarithmic molar fraction of hydrocarbon chain growth and has a linear dependence (Figure 1).

In practice, deviations from such dependence are quite commonly observed. This is the production of methane, which is formed in larger quantities. While ethane/ethene is formed in smaller amounts. Fischer–Tropsch synthesis is currently performed in two modes. These are the low temperature (LTFT) and high temperature (HTFT) modes. These modes differ in reaction conditions [25].

High-temperature FT synthesis (HTFT) most often occurs in the temperature range of 300–350 °C and a pressure of 2.5–4.5 MPa. Iron (Fe)-based catalysts producing mainly light products are used here. These gaseous and gasoline fractions contain large amounts of oxygenated hydrocarbon derivatives. Fluidized bed reactors and circulating catalyst reactors are used for high-temperature synthesis [27].

Low-temperature FT synthesis (LTFT) is most often performed in the temperature range of 200–240 °C and a pressure of 2.5 MPa. In this case, cobalt (Co) or iron (Fe) catalysts are used. These conditions cause the formation of products with a major proportion of n-alkanes with a long linear chain [28].

For the production of liquid fuels for diesel engines, it is therefore low-temperature FT synthesis that is preferable. Low-temperature synthesis is most often carried out in tubular reactors with a fixed catalytic bed, or in reactors with a catalyst in suspension [29].

The percentage of products may vary depending on the reaction temperature, pressure, and type of catalyst used. The difference in the composition of the primary product of low-temperature FT synthesis using iron (Fe) and cobalt (Co) catalysts is shown in Table 1.

When a cobalt (Co) catalyst is applied, the product contains more methane, fewer alkenes, and fewer oxygenates. This is due to the higher hydrogenation activity of the cobalt catalyst [30].

Low-temperature FT synthesis (LTFT) produces two types of major products. The difference between them is in the fractional composition and in the state under normal room conditions and atmospheric pressure. Secondary products are gases and water. Water may contain dissolved alcohols. It is important to separate secondary products from main products for further processing [31]. The lighter fraction, named as oil, is usually liquid and consists of hydrocarbons boiling up to about 370 °C. The heavier part, called wax, is usually solid, consisting mainly of high molecular weight n-alkanes [32]. The typical fractional composition of oil and wax produced by low temperature FT synthesis is given in Table 2.

Since Fischer–Tropsch synthesis catalysts are mostly used in powder form, reactions with these materials imply nanoeffects. Such phenomena occur with nanomaterials, for example, catalysts made totally or partially with nanostructured materials. Many sectors, for example, polymeric composite materials, are carrying out a lot of scientific and technological works, and there are also plans for a wide range of projects using nanomaterials. There has been tremendous interest in, and attention on, the promotion of nanostructured coatings. All this is because of the unique properties that are present, offering the possibilities of multifunctionality, a reduction in thickness, and a great spectrum of applications relating to technology.

However, recent works on nanoparticles show the potential risks of nanoparticle aerosol releases and allow a more balanced benefit/risk analysis [33]. For example, many studies highlight nanoparticle emissions due to coatings, paints [34], and tiles [35]. Cases of nanoparticle exposure in the field of occupational hygiene in coating workplaces have been reported [36]. These exposures can occur also when using powders [37,38,39,40].

From the point of view of the chemical composition of automotive fuels, they are therefore more suitable for the production of high-quality diesel than motor gasoline. Fluid catalytic cracking or catalytic hydrocracking is a suitable process for processing waxes to reduce molecular weight and improve low-temperature properties [42]. After treatment by selective hydroisomerization, high-boiling FT products can be used as high-quality lubricating oils [43].

The production of Fischer–Tropsch diesel on an industrial scale, however, inherently carries with it the risk and uncertainties associated with the economic impact. The risk assessment of such a process will include parameters such as CAPEX, costs related to industrial inputs, labor, input materials, and changes in the price of electricity, fuel, carbon allowance, etc. [44].

## 2. Materials and Methods

To determine the effect of FT-diesel distillation fraction on winter fossil diesel, mixtures with working names were selected:

- 100 vol. % pure fossil diesel (Diesel).
- 7 vol. % FT-diesel distillation fraction and 93 vol. % fossil diesel (FT7).
- 15 vol. % FT-diesel distillation fraction and 85 vol. % fossil diesel (FT15).
- 30 vol. % FT-diesel distillation fraction and 70 vol. % fossil diesel (FT30).
- 50 vol. % FT-diesel distillation fraction and 50 vol. % fossil diesel (FT50).
- 70 vol. % FT-diesel distillation fraction and 30 vol. % fossil diesel (FT70).
- 100 vol. % pure FT-diesel distillation fraction (FT100).

Pure fossil diesel was produced by Unipetrol RPA and it is fully compliant with standard EN 590+A1 for winter grade F and without FAME as a biocomponent. The FT-diesel distillation fraction (BP 180–360 °C) was produced in an atmospheric/vacuum distillation column in UniCRE laboratories from FT products created within the COMSYN project [45].

Distillation of FT products was performed in the laboratory distillation apparatus PILODIST. The PILODIST 105 distillation apparatus is a system with 70 theoretical plates and a Sulzer EX column packing. Atmospheric distillation and vacuum distillation of crude oil, petroleum products, and similar materials in nature can be performed on this unit. Atmospherically, the unit can be distilled up to 200 °C. Subsequently, a certain degree of vacuum must be included in the process. The entire distillation system is connected to the control software DCD4001.

To identify the fuel properties of the mixtures, the physiochemical properties of FT-diesel fraction were determined. In the evaluation of fuel density, kinematic viscosity, cetane number, cetane index, flash point, cold flow properties, lubricity, water content, PAU content, sulfur content, carbon content, hydrogen content, nitrogen content, higher calorific value, and distillation curves were measured. These fuel properties were compared for fuels containing volumetric amounts of different FT-diesel fractions.

The elemental analysis of each sample was carried out by the elemental analyzer FLASH 2000 according to the standard ASTM D5291 (The American Society for Testing and Materials: Standard Test Methods for Instrumental Determination of Carbon, Hydrogen, and Nitrogen in Petroleum Products and Lubricants).

The nitrogen and sulfur content in microscale (ppm) was measured by the Trace SN Cube Instrument according to the standards ASTM D5453 and ASTM D4629 (The American Society for Testing and Materials—Standard Test Method for Determination of Total Sulfur in Light Hydrocarbons, Spark Ignition Engine Fuel, Diesel Engine Fuel, and Engine Oil by Ultraviolet Fluorescence, and the Standard Test Method for Trace Nitrogen in Liquid Hydrocarbons by Syringe/Inlet Oxidative Combustion and Chemiluminescence Detection).

The heat of combustion was measured by LECO AC600 Semi-Automatic Calorimeter which provides calorific results for various organic materials, including coal, coke, fuel oils, and waste materials (DIN 51900-2: Determining the gross calorific value of solid and liquid fuels using the isoperibol or static-jacket calorimeter, and calculation of net calorific value).

The density was determined according to the standard ISO 12185:1996 (Crude petroleum and petroleum products—Determination of density—Oscillating U-tube method).

The kinematic viscosity was determined according to the standard ISO 3104:1994 (Petroleum products—Transparent and opaque liquids—Determination of kinematic viscosity and calculation of dynamic viscosity). The cetane index was determined according to the standard ISO 4264:2018 (Petroleum products—Calculation of cetane index of middle-distillate fuels by the four-variable equation). The cetane number was determined according to the standard ASTM D7668–17 (Standard Test Method for Determination of Derived Cetane Number (DCN) of Diesel Fuel Oils—Ignition Delay and Combustion Delay Using a Constant Volume Combustion Chamber Method). The distillation curve trend according to the standard ISO 3405:2011 (Petroleum products—Determination of distillation characteristics at atmospheric pressure). The cold flow properties were determined according to the standard EN 116:2015 (Diesel and domestic heating fuels—Determination of cold filter plugging point—Stepwise cooling bath method). Flash point was determined according to the standard ISO 2719:2016 (Determination of flash point—Pensky–Martens closed cup method). The lubricity was determined according to the standard ISO 12156-1:2018 (Diesel fuel—Assessment of lubricity using the high-frequency reciprocating rig (HFRR)—Part 1: Test method).

An analytical method for determining the FT-diesel fraction in diesel was also validated and performed using GC × GC-MS TOFMS. The percentage of the peak area was calculated from the measured amounts of the individual substances in the resulting mixtures. The sample is divided into individual components using two capillary columns (1 dimension—non-polar column, second dimension—polar column). The separation itself occurs by continuously repeated reinjection of the eluent from the first column to the second column by means of a two-stage thermal modulator.

The individual components are then detected by a mass detector, in particular a time-of-flight analyzer (TOFMS) in the gas phase under vacuum, according to the length of the flight time from the ion source to the detector. The samples were stored in dark brown glass bottles with a volume of 2 L. No dilution was required to perform a successful measurement.

For the measurement, a LECO PEGASUS^®^ BT 4D GC × GC-TOFMS was used. This machine assembly comprises a mass spectrometer with TOF flow analyzer in conjunction with GC × GC—two-dimensional gas chromatography. The two-dimensional gas chromatograph is equipped with a primary fused silica capillary column Rxi-5Sil MS and a secondary fused silica capillary column Rxi-17SilMS. The identification of individual substances was performed by comparing the measured spectrum with the National Institute of Standards and Technology (NIST) library. Device parameters and measurement conditions are shown in Table 3. For the statistical evaluation and graphical representation of the results, Matlab R2015a and R 4.0.2 were used.

Parameters were always measured three times and results represent the average value from the three measurements with the expanded uncertainty with 95% confidence interval. The expanded uncertainty *U* of the measurand was obtained by multiplying the combined standard uncertainty *u(y)* by a coverage factor *k*, which gives the best estimate of the value attributable to the measurand. The value of the coverage factor *k* was chosen to meet the probability of coverage of about 95%, which for a normal distribution, corresponds to the factor *k* = 2.

## 3. Results

### 3.1. Fuel Parameters

**Figure 2:**
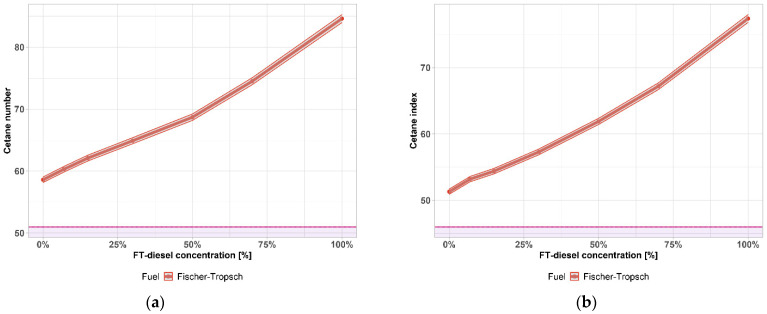
(**a**) Cetane number of FT-diesel mixtures; (**b**) cetane index of FT-diesel mixtures.

Figure 2a,b shows the change in cetane number and cetane index as a function of the FT-diesel concentration. The cetane number characterizes the ability of diesel to ignite, otherwise called reactivity. The reactivity is manifested by a delay between the injection of fuel into the cylinder and the increase in the pressure in the cylinder, after the ignition of the mixture, to a maximum value. The greater the reactivity of the fuel, the more regular and perfect its combustion and consequently the operation of the engine.

The minimum value of the cetane number according to the requirements of the EN 590 standard is 51, the minimum value of the cetane index according to the same standard is 46—both shown by the purple area. A significant increase of both cetane number and cetane index is shown with the addition of FT-diesel to conventional diesel. The gray area represents the 0.76% accuracy of the measurement.

The highest cetane number has n-alkanes (paraffins), for which the number further increases with chain length. The cetane number decreases from n-alkanes to aromatics, depending on the number of substituents, their length, branching, double bond position, etc. The more branched the hydrocarbon chains, the lower the cetane number. The medium cetane number is for cycloalkanes and alkylbenzenes, the lowest cetane number is for alkylnaphthalenes.

The minimum limit of the cetane number for a cold start is 40 units, for an easy cold start, at least 50 units. As shown in Figure 2, FT-diesel is a high cetane fuel based on a hydrocarbon composition.

As the test on the engine is relatively demanding, the cetane index was later introduced as a characteristic of the ignition ability (Figure 2b). The cetane index can be determined on the basis of a calculation from the results of laboratory tests of density and distillation (values of temperatures at which 10%, 50% and 90% of the tested fuel were distilled). The cetane index is not the same for the same fuel as the cetane number, practically, it is always a few units lower.

**Figure 3:**
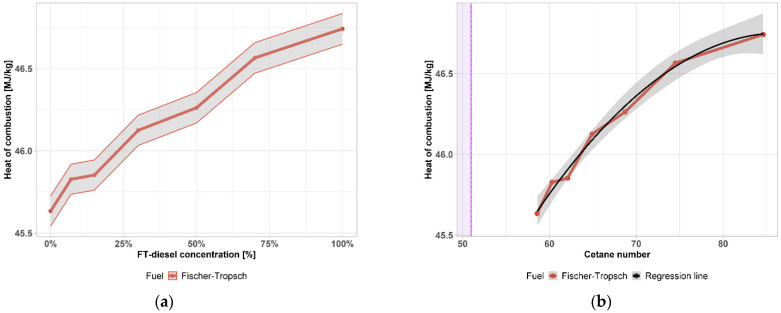
(**a**) Heat of combustion of FT-diesel mixtures; (**b**) regression function of heat of combustion as a function of cetane number.

A further measured property is the heat of combustion, which is a heat released when some amount of a substance is completely burned. The heat of combustion of FT-diesel mixtures is depicted in Figure 3a, where the gray area represents the 0.2% accuracy of the measurement. This property is also closely tied to the cetane number. According to the measured data, there is a quadratic relation between the properties ($R_{adj}^{2}=0.987$, *p*-value = $7.569^{-5}$):

(4) $$\Delta H_{c}^{{}^{\circ}}=46.14+0.96\cdot CN-0.24\cdot CN^{2}$$

This relation between properties is shown in Figure 3b. The gray area is the 95% confidence interval.

**Figure 4:**
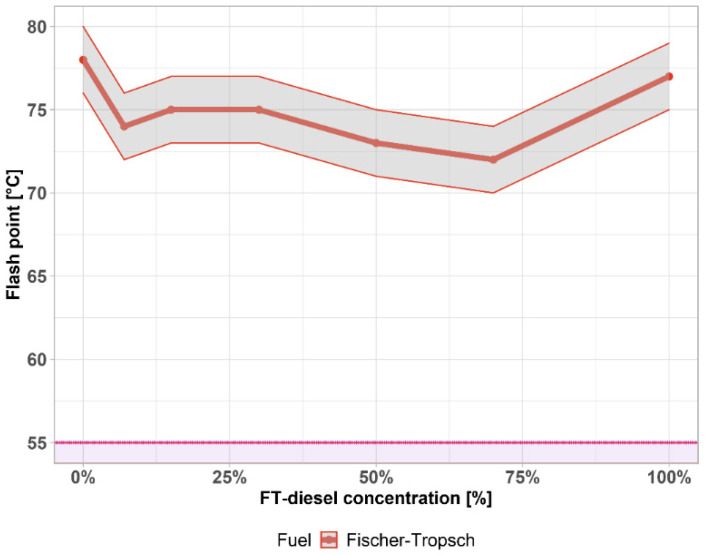
Flash point of FT-diesel mixtures.

Another important measured parameter is the flash point. The flash point values of diesel are usually between 58 °C and 75 °C. The flash point of winter diesel is lower than in summer classes. Flammable liquids are classified in a hazard class. According to the EN 590, the minimum value of the flash point of diesel has to be higher than 55 °C, which characterizes it as a Class II, shown by the purple area. The values of the flash point of pure diesel are usually between 58 °C and 75 °C. Results in Figure 4 show that the FT-diesel in conventional diesel does not change the flash point much so that the values are above the limit in the whole range of measured concentration. The gray area represents the 1 °C accuracy of the measurement. The flash point is important for safety and determines the values that are required for handling, storage, transport, and classification, but at these temperatures (Figure 4), does not affect the combustion process and engine properties.

According to the cold flow properties, a distinction is made between the types of diesel sold and are decisive for the use and operability of diesel in winter. The cooling parameters are determined by the defined cooling of the diesel sample in the device, where the diesel periodically passes through a system of screens. The eliminated paraffins gradually clog the sieves and increase the pressure difference before and after the sieves. The precipitated paraffins gradually clog the sieves and increase the pressure difference before and after the sieves. The temperature at which a given pressure difference is reached is the desired cold filter plugging point (CFPP). The loss of filterability, cold filter plugging point (CFPP) was not characterized by low temperature fuel parameters and serviceability, especially in winter. After this temperature has been reached, although the diesel is pumpable and possibly even starting the engine, it goes out ten times after a while to see if such a thick layer of solidified paraffin has formed on the filter, while the liquid fraction of the diesel does not pass through sufficiently. The temperature of loss of filterability must be determined by the cooling parameters and determined by the temperature at which the diesel is usable.

Although the middle distillates derived from the FT synthesis have an excellent cetane number, they do not meet the requirements for low-temperature properties, which results from their n-alkane character. Paraffins (n-alkanes) are solids dissolved in diesel under normal conditions, but with a decreasing temperature, they begin to return to the solid state and prevent the transport of fuel to the engine.

It is therefore common to use FT synthesis to produce higher molecular weight products (waxes) which are subsequently processed by conventional refining technologies, such as hydrocracking.

**Figure 5:**
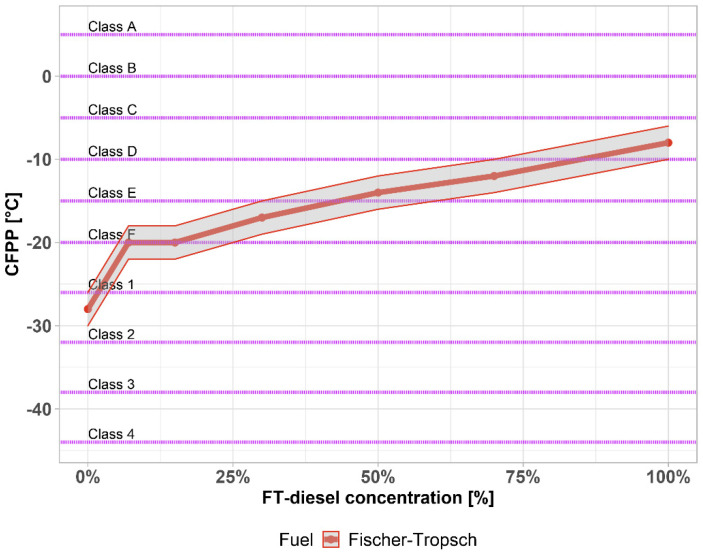
Cold filter plug point of FT-diesel mixtures.

Increasing the concentration of FT-diesel also increases the cold filter plug point. Figure 5 shows the CFPP with 1 °C accuracy, together with the highlighted climate-related requirements on diesel fuels. Base diesel fuel complies with all temperate climates and Class 0 and 1 of arctic or severe winter climates. The addition of FT-diesel also increases the temperature of CFPP. Class F is not satisfied around 15%, Class E around 50%, and Class D around 85%.

The density of the fuel is mainly determined by the content of aromatics. It affects the calorific value of the fuel, which is related to the type of hydrocarbons. Density is also of commercial importance in fuel supplies where it is used for volume-to-weight conversions and vice versa. Density is also used to calculate the cetane index and also affects the reactivity of diesel.

The effect of diesel density on engine power results from the fact that the injection pump operates by volume, and thus the amount of fuel injected increases with its specific gravity. It is stated that an increase in density of 0.01 g per 1 mL of diesel will increase engine power by 0.4% to 1.6%. Specific fuel consumption decreases with increasing density and vice versa.

**Figure 6:**
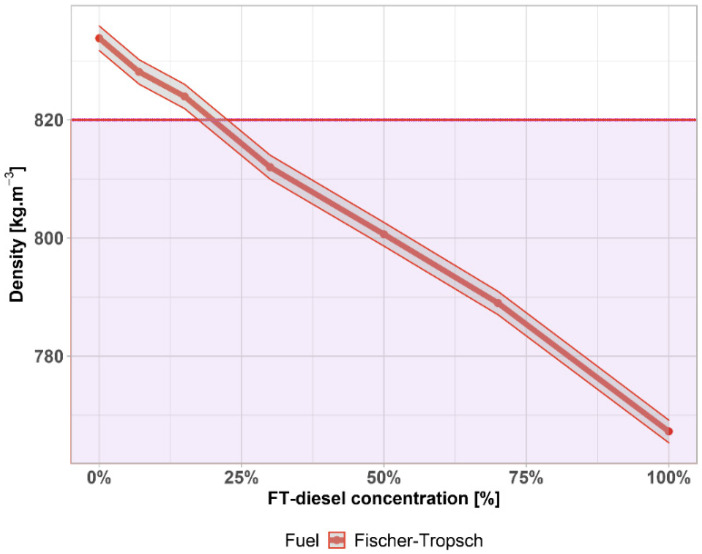
Density of FT-diesel mixtures.

In the case of the density of FT-diesel (in Figure 6), there is a significant linear drop going under the limit, the lowest admissible density is 820 kg·m^−3^ at 15 °C—shown by the purple area. This limit is exceeded around 21%. The gray area represents the 0.25% accuracy of the measurement.

When the density parameter is decreased, it is necessary to monitor the lubricity of the fuel. With a decreased density, no lubricating layer is formed on the moving parts and they are excessively worn. Conversely, at high fuel density, the formation of the mixture deteriorates due to insufficient fuel atomization and the proportion of unburned hydrocarbons, soot, and carbon monoxide increases, which externally manifests itself, especially in acceleration mode and full power mode as increased engine smoke (black smoke).

Another parameter is the kinematic viscosity. Viscosity is a measure of fuel flow and has some effect on lubricity. The viscosity of the fuel has a significant effect on the size of the droplets of fuel injected into the cylinder. Low viscosity has a positive effect on aerosol formation when injecting diesel into the combustion chamber. If the viscosity is high, it does not reach perfect dispersion, it can also lead to a deterioration in the pumpability of the diesel and a deterioration in the passage of the filters. Diesel that is too viscous is also the cause of carbon formation, as not enough fine aerosol is formed in the cylinder. A larger deviation of viscosity from higher values can cause poorer combustion in low pressure injection systems, resulting in a loss of performance, increased fuel consumption, and increased harmful emissions.

**Figure 7:**
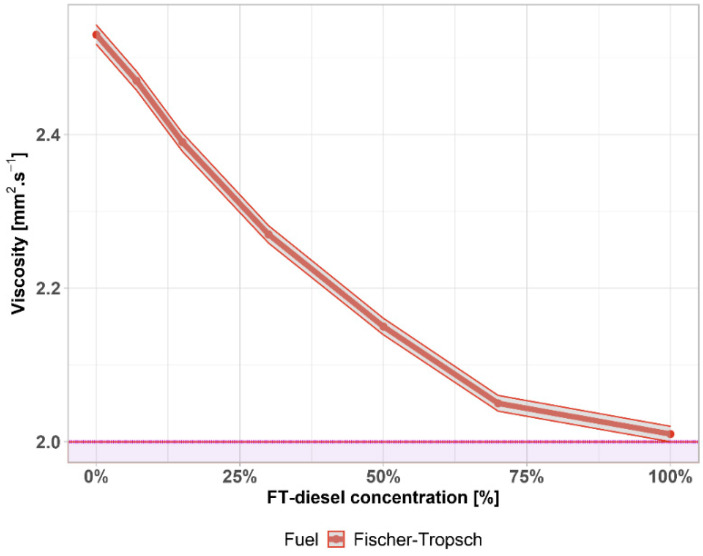
Viscosity of FT-diesel mixtures.

According to the results of kinematic viscosity depicted in Figure 7, the influence of FT-diesel is quite significant. The requirements given in EN 590 defines limits between 2.0 to 4.5 mm^2^·s^−1^—the lower limit is shown by the purple area. FT-diesel is slightly above this limit for the pure substance. The gray area represents the 0.25% accuracy of the measurement.

**Figure 8:**
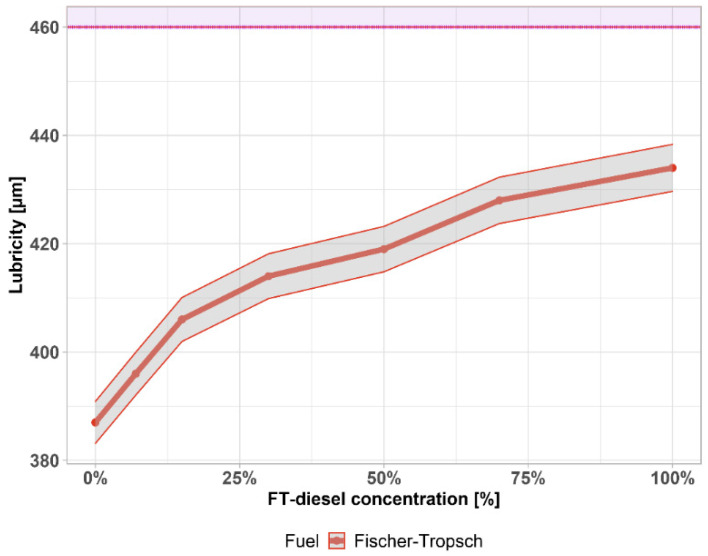
Lubricity of FT-diesel mixtures.

Lubricity is an important property of diesel that is necessary to ensure the proper functioning of fuel pumps and injectors. If diesel contains too many light components, there is a risk of damage to the moving parts of the fuel system due to reduced lubricity. The lubricity of FT-diesel is shown in Figure 8 with the 1% accuracy of the measurement shown by the gray area. According to the standard, the maximum permissible area diameter is 460 µm—shown by the purple area. FT-diesel satisfies this requirement in the whole interval.

**Figure 9:**
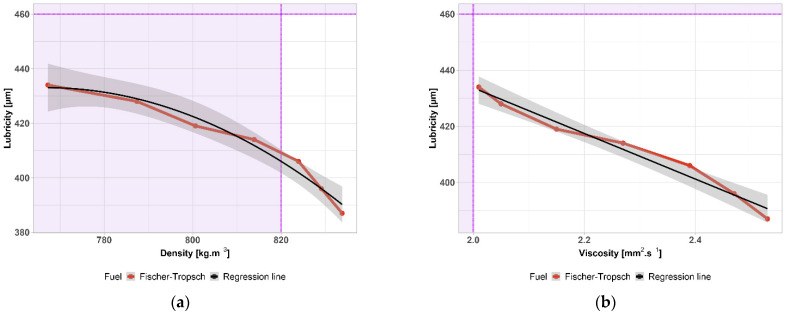
(**a**) Regression function of lubricity as a function of density; (**b**) regression function of lubricity as a function of viscosity.

The standard prescribes the minimum lubricity of diesel through the diameter of the wear surface, which is created by the friction of a vibrating ball on a metal surface to test the diesel environment at 60 °C. The better the lubricity of the diesel, the smaller the wear area. The maximum permissible surface diameter is 460 µm. Lubricity is closely related to density and viscosity. A strong linear correlation was found for both physical properties in the whole range of concentrations. Linear regression curves between their properties are shown in Figure 9. The gray area is the 95% confidence interval. The purple area highlights the limit exceeded, given by the standard. The important information derived from the measurement is that, even for a pure FT-diesel sample, it is not necessary to add lubricating additives in terms of lubricity characteristics, and a sufficient lubricating layer adheres to the moving parts of the fuel system despite the reduced density and viscosity at the lower limit of the diesel standard according to EN 590. Table 4 contains parameters and statistical significance.

### 3.2. Distillation Properties

The determination of the distillation curve is the dominant test that must always be performed when assessing the quality of diesel fuel. In order for the fuel to burn in the cylinder, it must evaporate and mix with air during injection. A sufficiently fine atomization of the fuel is necessary (small droplets have a larger total surface area and a higher evaporation rate), as well as a certain proportion of easily evaporable components. The fuel should be of the best possible composition and should not evaporate too rapidly after injection into the cylinder and therefore operate irregularly.

**Figure 10:**
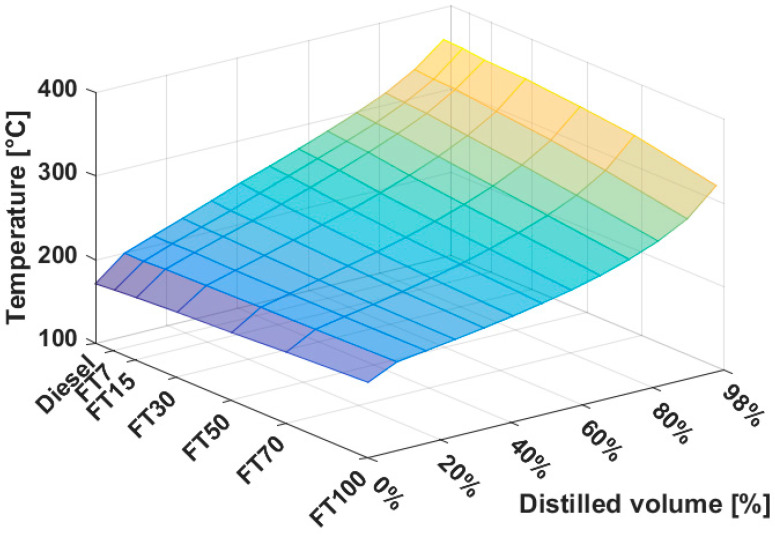
Distillation curve of FT-diesel mixtures.

As can be seen from Figure 10, FT-diesel does not behave significantly differently to conventional diesel. Generally speaking, the addition of the FT-diesel flattens the distillation curve. The start of a distillation begins at a higher temperature and ends at a lower temperature. This trend is gradual throughout the interval without any fluctuations or the presence of azeotropes.

Thus, depending on the course of the distillation curve, the fuel is light enough to gradually evaporate everything starting with the lightest fractions, and at the same time, regularly so that the combustion is uniform. Simultaneously, it contains heavier components, which evaporate gradually during the compression stroke, when they cool the walls of the combustion chamber.

### 3.3. Fuel Composition Characteristics

The chemical composition of both FT-diesel and conventional diesel is fundamentally the same. It consists of alkanes, cycloalkanes, alkenes, monoaromatics, diaromatics, and traces of sulfur, nitrogen, and water. Using the GC × GC-TOFMS system, the detail composition of fuels was measured. The results are in Table 5.

**Figure 11:**
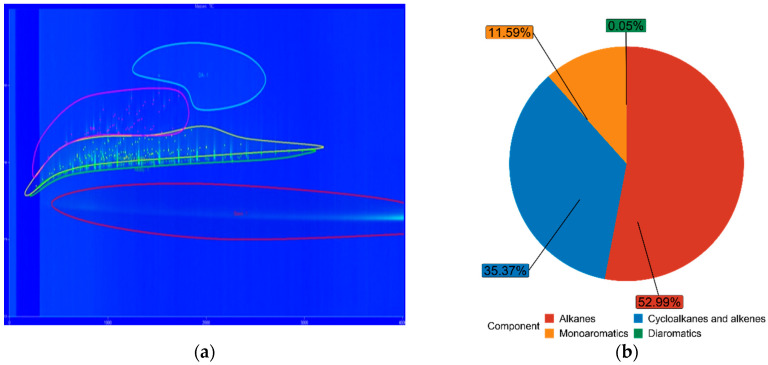
(**a**) Output of GC × GC-TOFMS for pure conventional diesel; (**b**) hydrocarbon composition of pure diesel.

**Figure 12:**
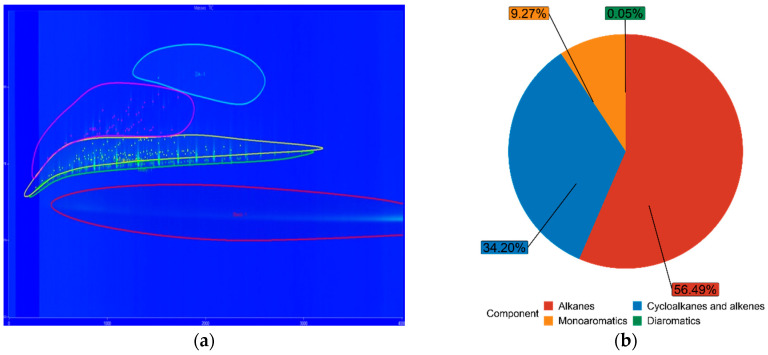
(**a**) Output of GC × GC-TOFMS for 7% FT-diesel; (**b**) hydrocarbon composition of 7% FT-diesel.

**Figure 13:**
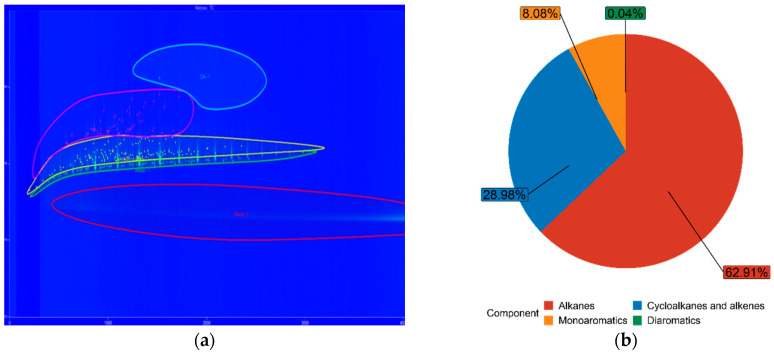
(**a**) Output of GC × GC-TOFMS for 15% FT-diesel; (**b**) hydrocarbon composition of 15% FT-diesel.

**Figure 14:**
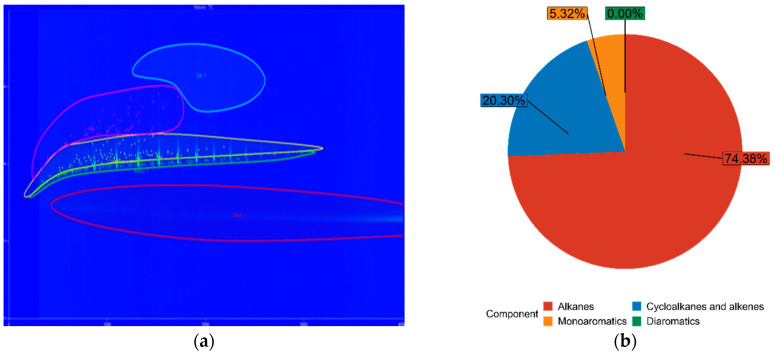
(**a**) Output of GC × GC-TOFMS for 30% FT-diesel; (**b**) hydrocarbon composition of 30% FT-diesel.

**Figure 15:**
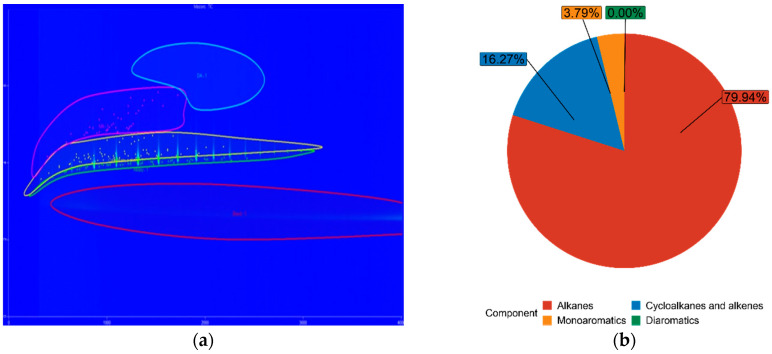
(**a**) Output of GC × GC-TOFMS for 50% FT-diesel; (**b**) hydrocarbon composition of 50% FT-diesel.

**Figure 16:**
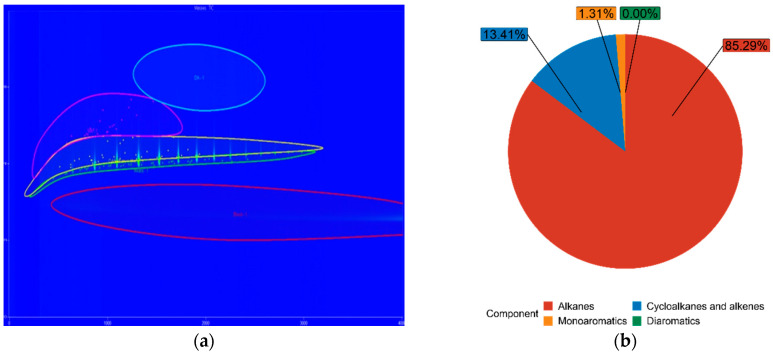
(**a**) Output of GC × GC-TOFMS for 70% FT-diesel; (**b**) hydrocarbon composition of 70% FT-diesel.

**Figure 17:**
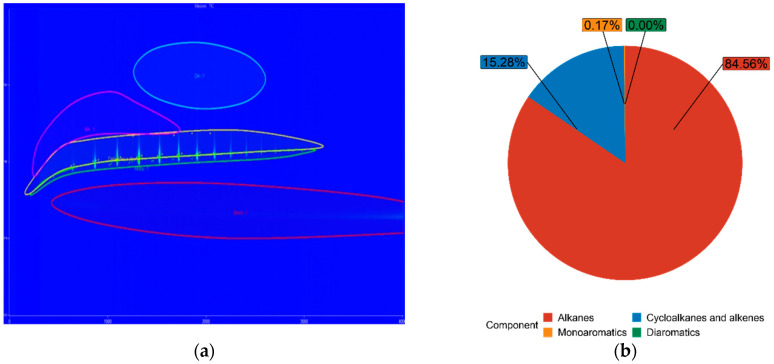
(**a**) Output of GC × GC-TOFMS for pure FT-diesel; (**b**) hydrocarbon composition of pure FT-diesel.

The blue figures are output images from GC × GC-TOFMS, where the green area represents found alkanes, yellow are cycloalkanes and alkenes, pink are monoaromatics, and blue are diaromatics. Figure 11 depicts pure diesel, Figure 12 depicts 7% FT-diesel mixture, Figure 13 depicts 15% FT-diesel mixture, Figure 14 depicts 30% FT-diesel mixture, Figure 15 depicts 50% FT-diesel mixture, Figure 16 depicts 70% FT-diesel mixture, and Figure 17 depicts pure FT-diesel.

## 4. Discussion

Ra et al., 2021 also investigated the Fischer–Tropsch diesel fuel produced by coal liquefaction. They measured (among others) the T90 of distillation curve, CFPP, flash point, lubricity, viscosity, cetane index, and density. This high paraffinic diesel complying with the EN 15940:2016 expectedly shows higher values of measured parameters, since in our research, FT-diesel was used with a lower concentration of paraffins. The fuel produced by Ra et al. is more appropriate for use in a warmer climate (CFPP > 0 °C, viscosity 3.4 mm^2^ s^−1^). One of the advantages would be a lower fuel consumption [46].

Sajjad et al., 2015 provided a comparative analysis of diesel blends together with FT20 in the context of fuel properties, combustion, engine performance, and emission characteristics. Pure FT-diesel had almost identical properties, such as density, heat of combustion, and cetane number. Viscosity, however, was approx. 0.75 mm^2^ s^−1^ higher. The obtained physicochemical properties of the FT20 mixture are comparable with our FT15 mixture. The difference in the density and heat of combustion is just 1% and 2% lower, respectively. Since Sajjad et al. used diesel fuel with a higher viscosity and a lower cetane number, consequently, this mixture also had the same shift in these parameters [47].

Du et al., 2014 experimentally investigated the effect of FT-diesel blends on combustion and particle size distribution, together with the evaluation of physicochemical properties, such as density, cetane number, low heating value, and T90. Both conventional diesel and FT-diesel used by Du et al. contained a higher percentage of aromatics and a lower percentage of paraffins. Consequently, the density of the mixtures was higher and the octane number with heat of combustion were lower. Nevertheless, the trend of the latter was the same, so the behavior of the mixtures is qualitatively similar [48].

Similar research was done by Parravicini et al., 2021, for FT20 (difference in heat of combustion ~5%, density ~1%, and cetane number ~2%) [49], Sadeq et al., 2021, for FT50 (difference in density ~2%, cetane number 10%, and heat of combustion ~1%) [50], and Schaberg et al., 2005, for lower temperature fuels FT50, resp. FT 80 (difference in density <1%, resp. 4%; cetane number 11%, resp. 28%; flash point 5%, resp. 11%; viscosity 15%, resp. 27%; CFPP 22%, resp. 29%; heat of combustion 6%, resp. 8%; and lubricity 5%, resp. 7%) [51]. For completeness, it can be added that the lubricity is higher compared to the ethanol-diesel blend [52].

The distillation curve of both the pure and FT-diesel mixture was investigated as well. Schaberg et al., 2005, measured T10, T50, T95 and FBP for FT0, FT50, FT80, and FT100. Conventional diesel used by us contained a higher concentration of low volatile components, thus the distillation temperatures of all diesel mixtures have lower temperatures in T10 (avg. 5% lower) and T50 (avg. 9% lower) [51]. T95 and FBP were almost the same (~1% difference). The distillation curve for pure Fischer–Tropsch diesel was also measured by Gough and Bruno, 2012. A higher concentration of volatile compounds caused a higher distilled amount at lower temperatures [53]. This trend continued throughout the whole range of measured volumes.

Comprehensive two-dimensional gas chromatography coupled with mass spectrometric detection GC × GC-MS was successfully used for a composition determination of a FT-diesel mixture. This powerful technique was able to provide detailed information about the present chemicals and their volume. Lissitsyna et al., 2014 used this method for a PIONA analysis (determination of n-paraffins, iso-paraffins, olefins, naphthenes, and aromatics) of kerosene samples [54]. Westhuizen et al., 2010 used this method for the analysis of Fischer–Tropsch oils obtaining quantitative data of the region from C_7_ to C_20_ [55]. This analysis, however, is not often used for the evaluation of Fischer–Tropsch fuel blending and this article is one of a few (if not the first).

## 5. Conclusions

In this article, the blending of Fischer–Tropsch diesel with conventional diesel and its fuel parameters was discussed. The main discoveries are summarized below:

1. The physicochemical properties of Fischer–Tropsch diesel are almost comparable to conventional diesel. In properties such as cetane number/index and heat of combustion, synthetic fuels are even better;
2. FT-diesel is an advanced biofuel which can be produced from renewable energy sources and waste materials;
3. The fuel meets the sustainability criteria;
4. The fuel is without the negative effects of biodiesel in the form of FAME. It is a pure hydrocarbon fuel without sulfur or polyaromatic hydrocarbons. Emissions are cleaner and lower.

## Figures and Tables

**Table 1:** Composition of Low-temperature FT synthesis products [21].

Components	Fe [wt. %]	Co [wt. %]
Methane	4.3	5.6
Ethylene	1	0.1
Ethane	1.0	1.0
C_3_–C_4_ alkenes	6.0	3.4
C_3_–C_4_ alkanes	1.8	1.8
C_5_–C_10_ alkenes	7.7	7.8
C_5_–C_10_ alkanes	3.3	12.0
C_5_–C_10_ oxygenated compounds	1.3	0.2
C_11_–C_22_ alkenes	5.7	1.1
C_11_–C_22_ alkanes	13.5	20.8
C_11_–C_22_ oxygenated compounds	0.3	0.0
C_22+_ alkenes	0.7	0.0
C_22+_ alkanes	49.2	44.6
Alcohols (dissolved in water)	3.9	1.4
Carboxylic acid (in water)	0.3	0.2

**Table 2:** The typical fractional composition of oil and wax produced by LTFT synthesis [41].

Distillation Range [°C]	Oil [vol. %]	Wax [vol. %]
30−160 °C	44	3
160−270 °C	43	4
270−370 °C	13	25
370−500 °C	0	40
>500 °C	0	28

**Table 3:** Parameters of GC × GC-TOFMS.

Machine Assembly	LECO PEGASUS^®^ BT 4D GC × GC-TOFMS
Primary column	Rxi-5SilMS, 30 m × 0.25 mm, d = 0.25 µm
Secondary column	Rxi-17SilMS, 1.3 m × 0.15 mm, d = 0.15 µm,
Carrier gas	He, 1 mL/min
Temperature program	40 °C (1.5 min), 40–300 °C (4°C/min), 300 °C (0.5 min)
Injector	20–320 °C, 720 °C/min, Split 500:1
Sample volume	0.2 µL
Ion Source	250 °C
Modulation period	8 s

**Table 4:** Parameters of the regression equation $WSD=A+Bx+Cx^{2}$ depicted in Figure 6. Correlation coefficients, $R_{adj}^{2}$, and p-value for both physical properties.

Property	A	B	C	$R_{adj}^{2}$	*p*-Value
Density	412.000	−40.809	−10.661	0.9619	6.442 × 10^−4^
Viscosity	596.180	−81.239	0 *	0.9687	3.767 × 10^−5^

* Value was not statistically significant, so it was not included in the table and figure.

**Table 5:** Measured chemical composition of FT-diesel mixtures by GC × GC-TOFMS.

Mixture	Alkanes	Cycloalkanes and Alkenes	Monoaromatics	Diaromatics
Diesel	52.99%	35.37%	11.59%	0.05%
FT7	56.49%	34.20%	9.27%	0.05%
FT15	62.91%	28.98%	8.08%	0.04%
FT30	74.38%	20.30%	5.32%	0.00%
FT50	79.94%	16.27%	3.79%	0.00%
FT70	85.29%	13.41%	1.31%	0.00%
FT100	84.56%	15.28%	0.17%	0.00%

## Data Availability

Data is contained within the article.
